# Differences in travel burden between patients with self-reported curable and incurable cancer: results from a Dutch flash mob study

**DOI:** 10.1007/s00520-025-09675-4

**Published:** 2025-06-23

**Authors:** M. A. J. Versluis, E. C. S. de Boer, L. V. van de Poll-Franse, N. J. H. Raijmakers, P. A. J. Vissers, I. H. Dingemans, M. B. de Ruiter, M. Slingerland, A. K. L. Reyners, M. E. T. Tesselaar, A. N. M. Wymenga

**Affiliations:** 1 Graduate School of Social and Behavioral Sciences, Tilburg University, Tilburg, The Netherlands; 2 Research and Development, The Netherlands Comprehensive Cancer Organization (IKNL), Utrecht, The Netherlands; 3 Isala Oncology Center, Isala Hospital, Zwolle, The Netherlands; 4 Department of Medical and Clinical Psychology, Center for Research On Psychological and Somatic Diseases (CoRPS), Tilburg University, Tilburg, The Netherlands; 5 Department of Psychosocial Research and Epidemiology, The Netherlands Cancer Institute, Amsterdam, The Netherlands; 6 Department of Surgery, Radboud University Medical Center, Nijmegen, The Netherlands; 7 Dutch Federation of Cancer Patient Organisations (NFK), Utrecht, The Netherlands; 8 Dutch Federation for Medical Oncology (NVMO), Utrecht, The Netherlands; 9 Department of Medical Oncology, Leiden University Medical Center, Leiden, The Netherlands; 10 University Medical Center Groningen, University of Groningen, Groningen, The Netherlands; 11 Department of Gastro-Intestinal Oncology, The Netherlands Cancer Institute, Amsterdam, The Netherlands; 12 Medical Spectrum Twente, Enschede, The Netherlands

**Keywords:** Travel burden, Cancer, Oncological care, Palliative care, Flash mob study

## Abstract

**Purpose:**

To explore travel burden in patients with self-reported curable and incurable cancer.

**Method:**

A 2-day flash mob study was conducted in March 2023 among patients visiting medical oncology departments in 65 Dutch hospitals. Disease status was self-reported. Patients completed a questionnaire on travel time (one-way), travel problems, and willingness to travel. Descriptive analyses and logistic regression analyses were used to assess travel burden and its associated factors.

**Results:**

In total, 991 patients with curable and 1959 with incurable cancer were included. Patients with curable cancer more often reported daily or weekly hospital visits (63% vs. 22%, *p* < 0.001) and a travel time of less than 30 min (78% vs. 73%, *p* = 0.005). Almost one-third of patients with curable (28%) and incurable cancer (29%) experienced some travel problems. Patients with worse physical functioning and longer travel times were more likely to experience travel problems. Disease status was not associated with experiencing travel problems or the willingness to travel for oncological care. Instead, willingness to travel was associated with patients’ level of education, physical functioning, and tumour type.

**Conclusion:**

Being diagnosed with self-reported curable or incurable cancer was not associated with experiencing travel problems or the willingness to travel for oncological care. Experiencing travel problems was associated with physical functioning and travel time, and the willingness to travel was associated with level of education, physical functioning, and tumour type. To ensure accessible and patient-centred care, physicians should be aware of these potential barriers and aim to provide well-coordinated, personalised care close to home.

**Supplementary Information:**

The online version contains supplementary material available at 10.1007/s00520-025-09675-4.

## Introduction

Despite medical developments in cancer treatment, cancer remains the leading cause of death in the Netherlands [1]. In 2022, 126,987 patients were newly diagnosed with cancer, and more than 46,000 people died from cancer [2]. Although the symptom burden is well described, other factors may contribute to the total cancer burden. A recent Dutch study of patients with oesophageal, gastric, or pancreatic cancer who underwent surgery shows that travel distance may also contribute to the total cancer burden, either physically, socially, or financially [3]. In addition, a Dutch qualitative study reported that patients with advanced cancer and their informal caregivers identified the organisation of cancer care as an important factor, in which the scheduling of hospital visits should include patients’ personal preferences [4].

Several studies in patients with different types and stages of cancer have shown an association between increased travel burden and worse patient outcomes, such as delayed diagnosis, shorter survival time, and greater financial burden [5–8]. However, most of these studies have been conducted in less densely populated countries with different infrastructure and health care systems than the Netherlands. As the Netherlands is a densely populated country with an average of 533 people per square kilometre [9], geographical distance may be a less important contributor to travel burden, and other factors may be more important. However, these factors remain unexplored. For example, the distance may be relatively short, but heavy traffic may lead to longer travel times, and the inaccessibility of a hospital by public transport may also play a role. In addition, parking and public transport may be costly. Therefore, travel problems are not only defined by distance.

Travel problems may also be related to the treatment regimen. Cancer care is provided throughout the disease trajectory and may vary in intensity at different stages of the disease. The care provided depends on the goal of treatment, whether it is lifesaving or life-prolonging. Patients with curable cancer often receive more intensive treatment with the aim of curing the disease. In contrast, patients with incurable cancer often receive less intensive treatment, as quality of life and symptom management are the main goals of treatment. It is therefore likely that the travel burden experienced by these two patient groups may differ.

Descriptive analyses of Dutch medical oncology patients have already reported that 30% of patients experience travel problems [10]. However, more in-depth analyses are needed to explore which factors, including disease status, may be associated with the likelihood of experiencing these travel problems in order to reduce the total cancer burden and ensure equal access to oncological care. Therefore, this study is aimed at investigating the differences in travel burden between patients with self-reported curable and incurable cancer and assessing which factors are associated with experiencing travel problems in the Netherlands.

## Materials and methods

### Study design

A cross-sectional flash mob study was conducted in the medical oncology departments of 65 out of 72 (90%) Dutch hospitals on 13 and 14 March 2023. Patients completed a questionnaire about their travel problems, which could be completed on paper, on a tablet provided by the hospital, or on a private mobile phone via the Patient Reported Outcomes Following Initial treatment and Long‐term Evaluation of Survivorship (PROFILES) registry [11]. Patients were supported by a medical specialist or nurse if needed. In addition, patients who were scheduled for a telephone or video consultation with a health professional from the unit during these 2 days were also invited to complete the questionnaire, either by telephone or digitally. The flash mob study was exempted from ethical approval by the Medical Ethics Review Board of the University Medical Centre Groningen. Patients were informed about the aim of the study and that completing the questionnaire would be considered as consent to use their information for this research purpose.

### Study population

All patients aged 18 years and older who were diagnosed with a solid tumour, excluding lung cancer, and who had an appointment with a medical oncologist of the medical oncology department or oncology day care unit on 13 or 14 March 2023 were eligible for inclusion. All patients with self-reported cancer, both curable and incurable, were included in this analysis. Patients with unknown or missing disease status (*n* = 565 (13%)) or who reported that their cancer was cured (*n* = 822 (19%)) were excluded from this analysis.

### Measurements

#### Travel burden

Patients were asked to report their travel time in minutes for a one-way trip to the hospital, which was then categorised as < 30 min, 30–60 min, and > 60 min. Categorical questions were used to report how often they had to travel to the hospital, what mode of transport they used, and who they travelled with. Patients were asked if they experienced travel problems, which was answered on a 4-point Likert scale (no, sometimes, often, or always). When patients indicated that they sometimes, often, or always experienced travel problems, they were also asked which factors contributed most to these problems. The response options were feeling a burden to others, frequency of hospital visits, travel costs, feeling too ill or too tired, travel time, not having a vehicle, not being able to travel alone, having problems with getting a taxi or specialised care transport, and problems combining hospital visits with work. Finally, patients were asked what their maximum travel time was that they were willing to travel for their current treatment, a follow-up appointment, and for a hospital that was more specialised in their tumour type. The response options were ‘Less than half an hour’, ‘Between half an hour and 1 h’, ‘Between 1 h and 1,5 h’, ‘Between 1,5 h and 2 h’, ‘Between 2 and 3 h’, and ‘More than 3 h’. This was then categorised as < 30 min, 30–60 min, 60–90 min, and > 90 min. They were also asked which factors were important in deciding upon this maximum travel time (hospital specialised in their tumour type, availability of a particular treatment, whether someone could come with them to their appointment, ability to take part in a clinical trial, feeling too ill or too tired, travel distance, frequency of visits, availability of public transport, travel costs, and the ability to combine hospital visits with work). The latter was answered on a 4-point Likert scale ranging from ‘not at all’ to ‘very much’. Patients who answered ‘quite a bit’ or ‘very much’ were then categorised as ‘yes, this is an important factor’. An English translation of the Dutch questionnaire is included as a supplement (Appendix 7).

#### Socio-demographic and clinical characteristics

Age, sex, education level, tumour type, comorbidities, and disease status were self-reported in the questionnaire. Disease status was assessed by the following question: ‘What is your current disease status?’. Response options included ‘I (probably) no longer have cancer’, ‘I have cancer, but I can get better’, ‘I have cancer and cannot get better’, ‘My cancer is not going away, but is stable at the moment’, and ‘I do not know’. Patients were classified as curable if the answer was ‘I have cancer, but I can get better’ and as incurable if the answer was ‘I have cancer and cannot get better’ or ‘My cancer is not going away, but is stable at the moment’. Physical functioning was measured using the specific subscale of the European Organisation for Research and Treatment of Cancer Quality of Life Core Questionnaire (EORTC QLQ-C30) [12, 13]. The score was linearly transformed into a score ranging from 0 to 100, with higher scores indicating better physical functioning.

### Statistical analysis

Descriptive analyses were used to describe socio-demographic and clinical characteristics and travel burden in patients with curable and incurable cancer. Chi-squared tests and two-sample *t*-tests were used to assess differences in travel burden between these two patient groups. Multivariable logistic regression analyses were used to explore possible factors associated with experiencing travel problems and the willingness to travel for treatment, follow-up, or for a hospital specialised in their tumour type. In addition, sensitivity analyses were conducted by also performing all logistic analyses separately for breast cancer patients and all patients excluding breast cancer patients due to the overrepresentation of breast cancer patients in this study. All statistical analyses were performed using STATA version 17.0, and a *p*-value < 0.05 was considered statistically significant.

## Results

A total of 4337 patients were enrolled in the flash mob study, of whom 2950 (68%) had reported to currently have cancer and were included in this analysis (Appendix 1). Of these patients, 991 (34%) reported having curable cancer, and 1959 (66%) reported having incurable cancer. Sixty-four percent of patients with curable cancer and 39% of patients with incurable cancer were younger than 65 years (Table 1). The majority of patients with curable cancer were diagnosed with breast cancer (57%), whereas patients with incurable cancer were more diverse in terms of tumour type (23% breast, 17% prostate, and 16% colorectal). The mean physical functioning score was higher in patients with curable cancer (76 (SD 22)) than in patients with incurable cancer (70 (SD 24), *p* < 0.001).

**Table 1:** Socio-demographic and clinical characteristics stratified by incurable and curable cancer

	Patients with curable cancer (*n* = 991) *N* (%)^*^	Patients with incurable cancer (*n* = 1959) *N* (%)^*^
Sex		
*Male*	252 (25)	948 (48)
*Female*	736 (74)	1007 (51)
*Missing*	3 (0)	4 (0)
Age (in years)		
≤ *39*	260 (26)	179 (9)
*40–65*	380 (38)	586 (30)
*65–74*	241 (24)	673 (34)
> *75*	107 (11)	517 (26)
*Missing*	3 (0)	4 (0)
Education**		
*Low*	279 (28)	643 (33)
*Medium*	337 (34)	679 (35)
*High*	361 (36)	606 (31)
*Missing*	14 (1)	31 (2)
Tumour type		
*Breast*	566 (57)	457 (23)
*Prostate*	30 (3)	334 (17)
*Colorectal*	97 (10)	306 (16)
*Upper GI*	92 (9)	206 (11)
*Urology*	35 (4)	152 (8)
*Other*	171 (17)	504 (26)
*Missing*	6 (1)	4 (0)
Currently receiving cancer treatment *(%yes)*	859 (87)	1683 (86)
Receiving treatment for a condition other than cancer *(%Yes)*	178 (18)	448 (23)
Physical functioning *(0–100) (mean)*	76 (SD 22)	70 (SD 24)

* Percentages may not add up to exactly 100%, due to rounding

** Educational level is defined as low (Lower school, LTS, LHNO, domestic school, VMBO, LEAO, ULO, MULO/MAVO and three years of HBS), medium (MBO, MTS, MEAO, HAVO, VWO, HBS and MMS) and high (HBO and university)

### Travel-related characteristics

Patients with curable cancer reported shorter one-way travelling times (< 30 min: 78% vs. 73%, *p* = 0.005) and less often reported to be treated at an academic hospital than patients with incurable cancer (15% vs. 20%, *p* = 0.002) (Table 2). Patients with curable cancer visited the hospital more often than patients with incurable cancer, respectively, 63% versus 22% visited the hospital on a daily or weekly basis (*p* < 0.001). Most patients travelled by car (independently (42%) or someone else drove them to hospital (39%)). Other modes of transport included walking or cycling (7%), taxi (6%), or public transport (3%). Sensitivity analysis excluding patients diagnosed with breast cancer still showed significant differences in travel time, type of hospital, and frequency of visits between patients with curable and incurable cancer (Appendix 2). However, in this sensitivity analysis, patients with curable cancer were more often treated in an academic hospital than patients with incurable cancer, respectively, 28% vs. 23% (*p* = 0.012).

**Table 2:** Travel-related characteristics stratified by incurable and curable cancer

	Patients with curable cancer (*n* = 991) *N* (%)^*^	Patients with incurable cancer (*n* = 1959) *N* (%)*	*p*-value**
Current travel time (one-way)			**0.005**
≤ *30 min*	771 (78)	1422 (73)	
*30–60 min*	169 (17)	433 (22)	
≥ *60 min*	48 (5)	100 (5)	
*Missing*	3 (0)	4 (0)	
Type of hospital***			**0.002**
*Academic*	144 (15)	388 (20)	
*Teaching*	498 (50)	947 (48)	
*General*	349 (35)	624 (32)	
*Missing*	-	-	
Frequency of hospital visits			** < 0.001**
*Daily* or weekly	622 (63)	431 (22)	
Every 2–4 weeks	329 (33)	1152 (59)	
*Once every 3 months* or less	29 (3)	349 (18)	
*Missing*	11 (1)	27 (1)	
Travel mode****			** < 0.001**
*Independent*	416 (42)	989 (50)	
*Dependent*	539 (54)	911 (47)	
*Missing*	36 (4)	59 (3)	
Who usually accompanies you when visiting hospital? (multiple answers possible)			
*Nobody*	82 (8)	228 (12)	**0.005**
*Partner*	688 (69)	1315 (67)	0.234
*Child(ren)*	189 (19)	422 (22)	0.112
*Another family member*	78 (8)	87 (4)	** < 0.001**
*Friends or acquaintances*	130 (13)	157 (8)	** < 0.001**
*Other*	8 (1)	9 (0)	0.240
Problems with travelling			0.187
*None*	702 (71)	1354 (69)	
*Sometimes*	224 (23)	439 (22)	
*Often*	44 (4)	84 (4)	
*Always*	14 (1)	52 (3)	
*Missing*	7 (1)	30 (2)	

^*^Percentages may not add up to exactly 100% due to rounding. ^** ^Bold p-values indicate statistical significance (*p*<0.05), *** Type of hospital refers to the hospital they completed the questionnaire in. **** Travel mode was defined as independent (on their own: walking, cycling, or by car) or dependent (someone else drove them, public transport, or taxi services provided by the hospital)

### Travel problems

**Fig. 1:**
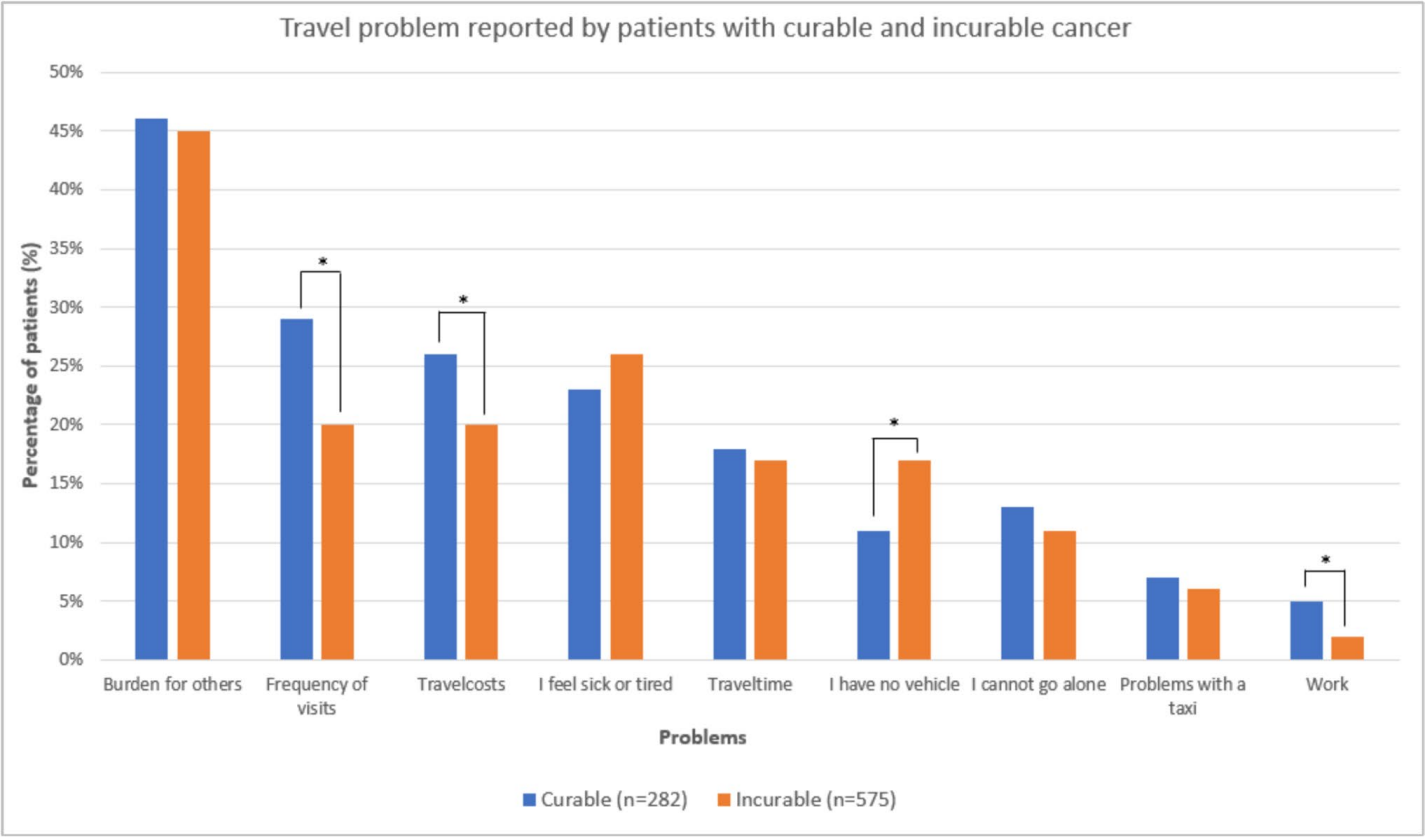
Type of travel problems of patients with cancer who reported to experience travel problems (*n* = 857). *Difference was statistically significant if *p* < 0.05

The majority of patients reported no problems with travelling for oncological care, which did not differ significantly between patients with curable (71%) and incurable cancer (69%). Among patients with curable cancer who experienced travel problems, 23% sometimes experienced travel problems, 4% often experienced travel problems, and 1% always experienced travel problems. Similarly, 22% of patients with incurable cancer sometimes experienced travel problems, 4% often and 3% always. The most common type of travel problem reported by the patients who sometimes, often, or always experienced travel problems was feeling like a burden to others, both for patients with curable (46%) and incurable (45%) cancer (Fig. 1). For patients with curable cancer, the other main problems were frequency of visits (28%) and cost of travel (25%). For patients with incurable cancer, the other main problems were feeling too ill or too tired to travel (25%), frequency of visits (20%), and travel costs (20%).

Multivariable analyses confirmed that being diagnosed with curable or incurable cancer was not significantly associated with experiencing travel problems (Table 3). Patients with better physical functioning scores (OR 0.82, 95% CI 0.79–0.85, *p* < 0.001), those currently receiving cancer treatment (OR 0.76, 95% CI 0.58–0.99, *p* = 0.039), and those who are treated in an academic hospital (OR 0.76, 95% CI 0.53–0.98, *p* = 0.035) were less likely to experience travel problems. Female patients (OR 1.38, 95% CI 1.09–1.77, *p* = 0.010), patients aged ≤ 39 years (OR 1.76, 95% CI 1.26–2.48, *p* = 0.001), patients with longer travel times (30–60 min: OR 1.90, 95% CI 1.52–2.37, *p* < 0.001, > 60 min: OR 3.06, 95% CI 2.03–4.61, *p* < 0.001), and patients who relied on others or public transport to get to the hospital (OR 1.32, 95% CI 1.09–1.59, *p* = 0.004) were more likely to experience travel problems. Sensitivity analysis showed that the results of the multivariable logistic regression analysis excluding patients diagnosed with breast cancer were comparable to those of the total population (Appendix 3). Among patients with breast cancer, only travel time and physical functioning remained significantly associated with experiencing travel problems.

**Table 3:** Multivariable logistic regression analysis of experiencing travel problems in cancer patients (*n* = 2629)

	Odds	95% CI	*p*-value
Sex			
*Male*	Ref	Ref	
*Female*	**1.38**	**1.08–1.77**	**0.010**
Age (years)			
≥ *75*	Ref	Ref	
*65–74*	**1.31**	**1.01–1.70**	**0.040**
*40–65*	**1.32**	**1.01–1.73**	**0.044**
≤ *39*	**1.76**	**1.26–2.48**	**0.001**
Disease status			
*Curable*	Ref	Ref	
*Incurable*	1.05	0.85–1.30	0.657
Education			
*Low*	Ref	Ref	
*Medium*	1.03	0.83–1.29	0.768
*High*	0.99	0.79–1.25	0.937
Receiving treatment			
*No*	Ref	Ref	
*Yes*	**0.76**	**0.58–0.99**	**0.039**
Physical functioning *(per 10)*	**0.82**	**0.79–0.85**	** < 0.001**
Tumour type			
*Colorectal*	Ref	Ref	
*Breast*	0.79	0.57–1.08	0. 139
*Prostate*	1.24	0.84–1.82	0.274
*Upper GI*	0.96	0.66–1.39	0.823
*Urology*	1.09	0.71–1.65	0.697
*Other*	0.92	0.68–1.26	0.616
Type of hospital			
*General*	Ref	Ref	
*Teaching*	1.02	0.83–1.24	0.869
*Academic*	**0.72**	**0.53–0.98**	**0.035**
Travel time (minutes)			
< *30*	Ref	Ref	
*30–60*	**1.90**	**1.52–2.37**	** < 0.001**
> *60*	**3.06**	**2.03–4.61**	** < 0.001**
Frequency of visits			
*Daily or weekly*	Ref	Ref	
*Every 2–4 weeks*	0.90	0.73–1.11	0.331
*Every 3 months or less*	0.80	0.58–1.11	0.181
Travel mode**			
*Independent*	Ref	Ref	
*Dependent*	**1.32**	**1.09–1.59**	**0.004**

^* ^Bold results indicate statistical significance (*p*<0.05), ^**^Travel mode was defined as independent (on their own: walking, cycling, or by car) or dependent (someone else drove them, public transport, or taxi services provided by the hospital)

### Willingness to travel

**Fig. 2:**
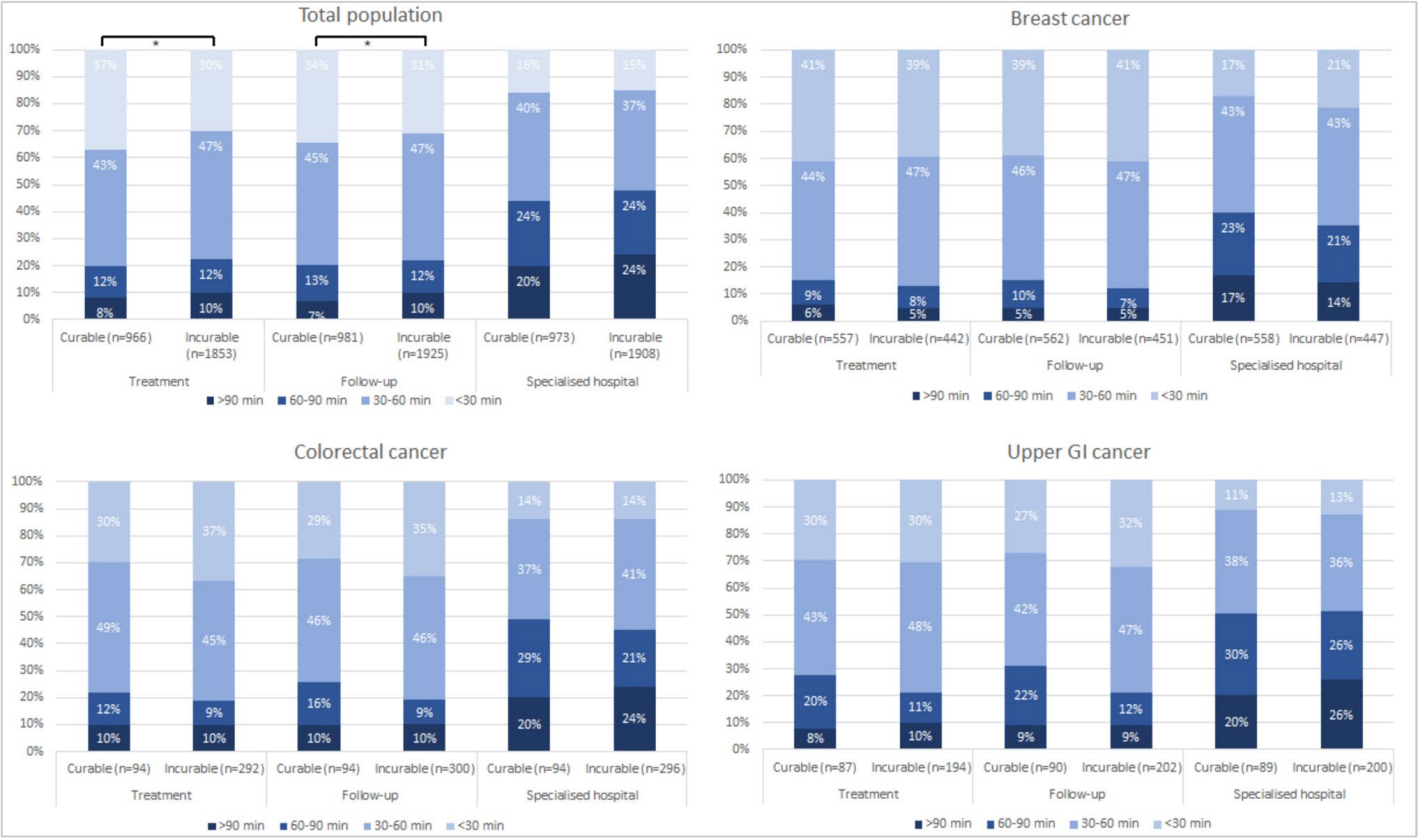
Maximum time that cancer patients are willing to travel (one-way) for their current treatment (*n* = 2819), a follow-up appointment (*n* = 2906), and for a hospital that is specialised in their tumour type (*n* = 2881), including tumour specific results (breast (*n* = 1023), colorectal (*n* = 403), and upper GI (*n* = 298)). *Difference was statistically significant if *p* < 0.05

**Fig. 3:**
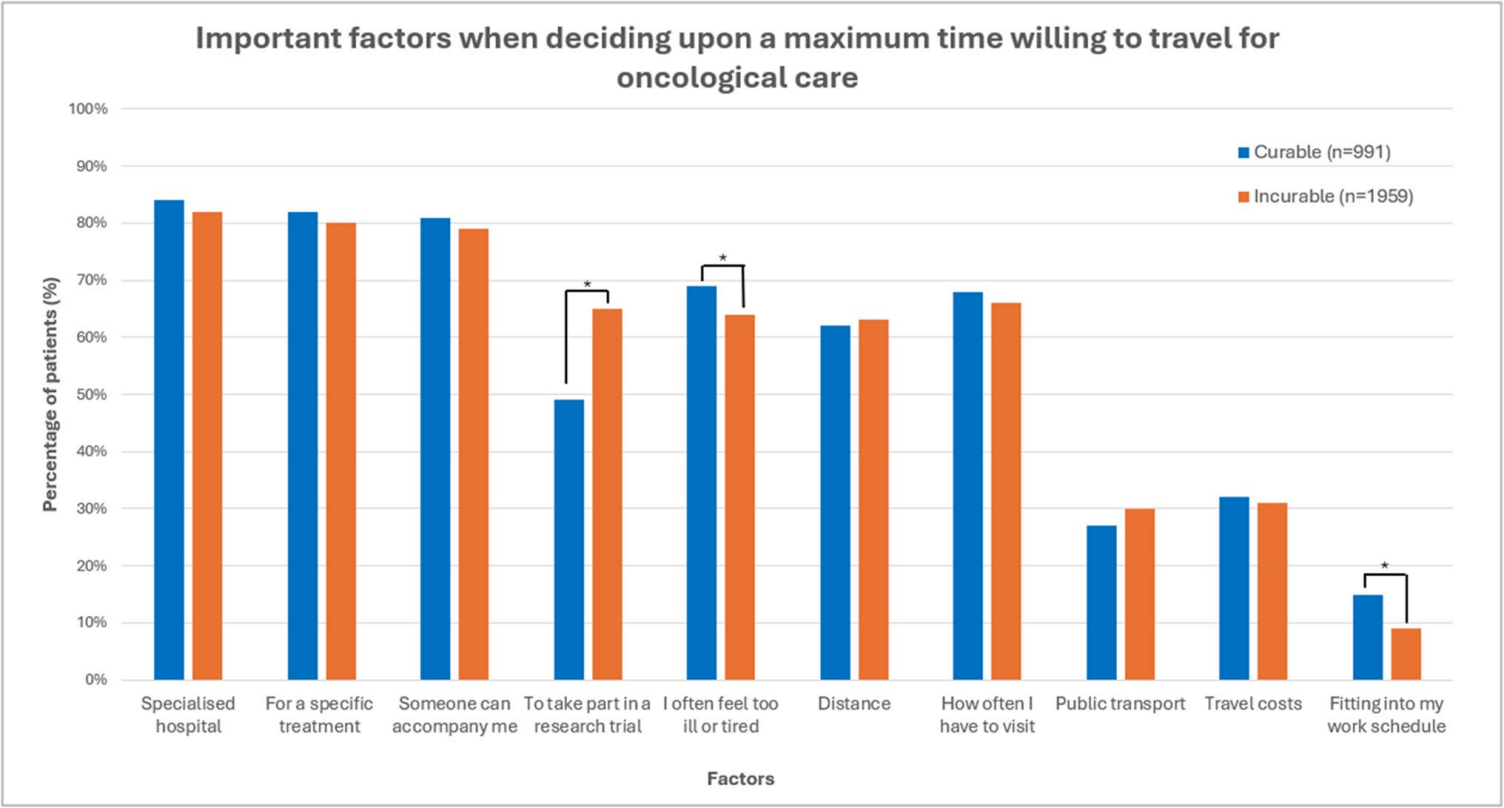
Important considerations when deciding on a maximum travel time for oncology care in curable and incurable cancer patients (*n* = 2950). *Difference was statistically significant if *p* < 0.05

Most patients were unwilling to travel > 60 min for their current treatment (80% for curable patients and 77% for incurable patients) and for their follow-up appointment (79% and 78%, respectively, Fig. 2). The percentage of patients with both curable and incurable cancer who were unwilling to travel > 60 min was lower for a hospital specialised in their tumour type; respectively, 56% and 52%. Stratified analyses showed no significant differences in willingness to travel between patients with curable cancer and patients with incurable cancer. Multivariable logistic regression analysis showed that patients with incurable cancer were more likely to be willing to travel > 30 min for treatment (OR 1.24, 95% CI 1.02–1.51, *p* = 0.03) than patients with curable cancer (Appendix 4). Being diagnosed with curable or incurable cancer was not associated with the willingness to travel for follow-up (Appendix 5) or a hospital specialised in their tumour type (Appendix 6). However, the association between disease status and the willingness to travel for treatment disappeared in the sensitivity analysis, in which patients diagnosed with breast cancer were excluded. A greater willingness to travel was primarily associated with a higher level of education and better physical functioning and varied by tumour type. For both curable and incurable patients, the two most important considerations in deciding their maximum travel time were the choice of a specialised hospital (84% and 82%, respectively) and a hospital offering a specific treatment not available elsewhere (82% and 80%, respectively, Fig. 3). In addition to these considerations, patients with curable cancer more often reported feeling too ill or too tired (69% vs. 64%, *p* = 0.007) and being able to fit in with their work schedule (15% vs. 9%, *p* < 0.001) as important considerations when deciding on their maximum travel time compared to patients with incurable cancer. Patients with incurable cancer often indicated that participation in a clinical trial was also an important factor in deciding their maximum travel time (65% vs. 49% of patients with curable cancer, *p* < 0.001).

## Discussion

This 2-day flash mob study in 65 Dutch hospitals showed that although most patients do not experience problems with travelling for oncological care, approximately one-third do (sometimes) experience travel problems. Patients with curable cancer more often reported to visit the hospital on a daily or weekly basis and more often reported shorter travel times than patients with incurable cancer. Being diagnosed with curable or incurable cancer was not associated with experiencing travel problems or the willingness to travel for oncological care.

Regardless of disease status, approximately 30% of patients sometimes experience problems travelling for oncological care with feeling like a burden to others being the most commonly reported problem. Moreover, 39% of patients reported that someone else drove them to hospital, and the multivariable regression analysis showed that patients who were dependent on others to get to the hospital were more likely to experience travel problems. This phenomenon could be described as the self-perceived burden on others and is well-known in patients with advanced cancer [14, 15]. It can be explained by the fact that they are more likely to become dependent on their informal caregivers as the disease progresses. However, our study suggests that this self-perceived burden is experienced not only by patients with advanced cancer but also by patients with curable cancer. This shows that patients with cancer, regardless of disease status, may experience travel problems to the same extent and that it is important to consider the travel burden in shared decision making. One of the other commonly reported travel problems for both patients with curable and incurable cancer was the cost of travel. In the Netherlands, patients receiving immunotherapy, chemotherapy, or radiotherapy are entitled to a travel allowance for the days of the treatment, including transport by taxi [16]. Though, the taxi sometimes must be shared with other patients which can lead to an increase in travel time. Additionally, patients who undergo treatment for 3 or more consecutive days may receive an accommodation allowance, eliminating the need for frequent travel. It is important that patients are aware of these entitlements and know how to apply for them.

Patients with curable cancer more often reported to visit the hospital on a daily or weekly basis but had shorter travel times than patients with incurable cancer. However, multivariable analysis showed no association between willingness to travel and disease status. We did find that patients with a higher level of education, patients with better physical functioning, and those who were diagnosed with a less common tumour type were more likely to be willing to travel > 30 min for oncological care. The latter was also reported in a previous Dutch study comparing willingness to travel between patients with rare cancers and patients with more common cancers, which showed that patients with rare cancers were more willing to travel as far as necessary to receive specialised care [17]. It is important to note that, in the Netherlands, oncological care for certain rare tumour types has been centralised, meaning that tumour-specific treatments are no longer available in every hospital, consequently resulting in longer travel times for patients. Conversely, our study indicates that patients diagnosed with the most common tumour type, breast cancer, are predominantly treated in non-academic hospitals. Despite the differences between tumour types, physicians should be aware of differences in patients’ willingness to travel and try to minimise travel time by providing well-coordinated and personalised care. For example, scheduling as many appointments as possible on the same day or replacing physical appointments with e-consultations or telephone calls could limit the frequency of hospital visits.

### Strengths and limitations

This 2-day flash mob study involved 65 out of 72 Dutch hospitals, with an overall response rate of 62%, making it highly representative of the Dutch cancer population. The representativeness is supported by the mean physical functioning scores in both patient groups, which are similar to those of other large cohort studies [18, 19]. Nevertheless, some limitations should be addressed. For example, the fact that patients with better health and higher educated patients are more likely to participate in a survey study may have led to (some) selection bias. However, this flash mob study was able to enrol a representative population in terms of educational level (almost one-third for each level), which is similar to the general Dutch population [20]. In addition, 57% of patients with curable cancer reported that they had been diagnosed with breast cancer, which is not consistent with national cancer data [21]. Patients with breast cancer are often younger patients who receive intensive treatment that requires them to be in hospital relatively often. They also tend to be more highly educated female patients who are more likely to participate in research. Frequent hospital visits and a higher propensity to participate in research may explain the over-representation of this tumour type in this study. It is also known that self-reported clinical data from patients are less reliable [22]. In this study, disease status was self-reported and may have led to an underestimation of patients with incurable cancer. Previous research has shown that patients are often unaware of their poor prognosis, even in the last few months before death [23, 24]. For example, in this study, 30 of the 364 (8%) patients with prostate cancer reported having curable cancer, whereas in the Netherlands, patients with prostate cancer usually only visit the oncologist for palliative systemic treatment. Therefore, a proportion of patients who reported having curable cancer may have been misclassified. Whether this remains similar or whether disease status has an influence on the likelihood of experiencing travel problems should be explored further with more detailed clinical data.

## Conclusion

There was no difference in experiencing travel problems between patients with self-reported curable and incurable cancer. Almost a third of all cancer patients (sometimes) experienced problems with travelling for oncological care, with feeling like a burden to others being the most commonly reported travel problem. Irrespective of disease status, patients with longer travel times and those with poorer physical functioning were more likely to experience travel problems. To prevent these travel problems from affecting the care a patient receives, it is important to consider travel burden during the shared decision-making process and to support patients in arranging travel allowance or accommodation allowance. In addition, the willingness to travel for oncological care was not associated with disease status but was related to the patients’ level of education, physical functioning, and tumour type. Physicians should be aware of potential travel problems and aim to provide patient-friendly well-coordinated, personalised care like combining hospital appointments on one day, digital consults when possible, care close to home, and providing information on travel allowance.

## Supplementary Information

Below is the link to the electronic supplementary material.

Supplementary file1 (DOCX 170 KB)

## Data Availability

No datasets were generated or analysed during the current study.
