# Supplementary material for: Differences in travel burden between patients with self-reported curable and incurable cancer: results from a Dutch flash mob study
Source: Support Care Cancer. 2025 Jun 23;33(7):610. doi: 10.1007/s00520-025-09675-4 (PMC12183130; doi:10.1007/s00520-025-09675-4)
Supplement: Supplementary file 1 — Supplementary file1 (DOCX 170 KB) [file 520_2025_9675_MOESM1_ESM.docx]

**Supplementary files**

**Appendix 1: flow chart**


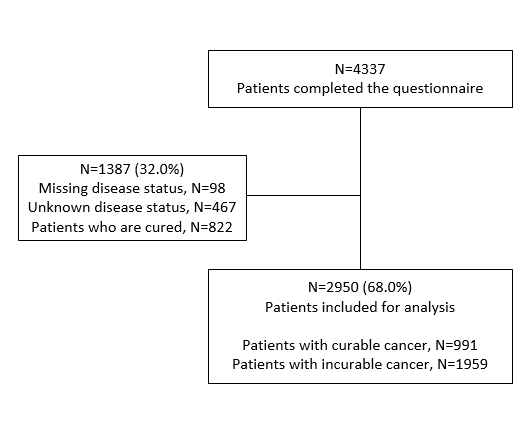


**Appendix 2: Univariate analysis of the travel characteristics of patients with curable and incurable cancer, stratified for breast cancer patients only and all other tumour types.**

|  | **Breast cancer** | | | **All other tumour types** | | |
| --- | --- | --- | --- | --- | --- | --- |
|  | Curable (n=566)  N(%) | Incurable (n=457)  N(%) | p-value | Curable (n=419)  N(%) | Incurable (n=1498)  N(%) | p-value |
| **Current travel time (one-way)** |  |  | **0.049** |  |  | **0.049** |
| *≤30 minutes* | 486 (86) | 367 (80) |  | 280 (67) | 1051 (70) |  |
| *30-60 minutes* | 70 (12) | 81 (18) |  | 98 (23) | 352 (24) |  |
| *≥60 minutes* | 8 (1) | 8 (2) |  | 40 (10) | 92 (6) |  |
| *Missing* | 2 (0) | 1 (0) |  | 1 (0) | 3 (0) |  |
| **Type of hospital**** |  |  | **0.034** |  |  | **0.012** |
| *Academic* | 24 (4) | 37 (8) |  | 118 (28) | 351 (23) |  |
| *Teaching* | 286 (51) | 224 (49) |  | 210 (50) | 720 (48) |  |
| *General* | 256 (45) | 196 (43) |  | 91 (22) | 427 (29) |  |
| *Missing* | - | - |  | - | - |  |
| **Frequency of hospital visits** |  |  | **<0.0001** |  |  | **<0.001** |
| *Daily* or weekly | 431 (76) | 99 (22) |  | 187 (45) | 332 (22) |  |
| Every 2-4 weeks | 113 (20) | 265 (58) |  | 214 (51) | 885 (59) |  |
| *Once every three months* or less | 18 (3) | 87 (19) |  | 11 (3) | 260 (17) |  |
| *Missing* | 4 (1) | 6 (1) |  | 7 (2) | 21 (1) |  |
| **Travel mode***** |  |  | **0.003** |  |  | 0.091 |
| *Independent* | 216 (38) | 221 (48) |  | 196 (47) | 767 (51) |  |
| *Dependent* | 326 (58) | 226 (49) |  | 211 (50) | 683 (46) |  |
| *Missing* | 24 (4) | 10 (2) |  | 12 (3) | 48 (3) |  |
| **Who usually accompanies you when visiting hospital?** (multiple answers possible) |  |  |  |  |  |  |
| *Nobody* | 56 (10) | 74 (16) | **0.003** | 55 (13) | 194 (13) | 0.930 |
| *Partner* | 401 (71) | 274 (60) | **<0.001** | 285 (68) | 1039 (69) | 0.581 |
| *Child(ren)* | 100 (18) | 105 (23) | **0.033** | 87 (21) | 316 (21) | 0.876 |
| *Another family member* | 55 (10) | 22 (5) | **0.003** | 22 (5) | 65 (4) | 0.428 |
| *Friends or acquaintances* | 96 (17) | 52 (11) | **0.012** | 33 (8) | 105 (7) | 0.547 |
| *Other* | 5 (1) | 4 (1) | 0.992 | 3 (1) | 5 (0) | 0.284 |
| **Problems with travelling** |  |  | 0.274 |  |  | 0.960 |
| *None* | 407 (72) | 310 (68) |  | 291 (69) | 1040 (69) |  |
| *Sometimes* | 128 (23) | 104 (23) |  | 95 (23) | 335 (22) |  |
| *Often* | 24 (4) | 18 (4) |  | 20 (5) | 66 (4) |  |
| *Always* | 6 (1) | 18 (4) |  | 8 (2) | 34 (2) |  |
| *Missing* | 1 (0) | 7 (2) |  | 5 (1) | 23 (2) |  |

* Percentages may not add up to exactly 100% due to rounding.

** Type of hospital refers to the hospital they completed the questionnaire in.

*** Travel mode was defined as independent (on their own: walking, cycling or by car) or dependent (someone else drove them, public transport or taxi services provided by the hospital).

**Appendix 3: Logistic regression analyses on experiencing travel problems.**

|  | Breast cancer (n=919) | | | All other tumour types (n=1710) | | |
| --- | --- | --- | --- | --- | --- | --- |
|  | Odds | 95% CI | p-value | Odds | 95% CI | p-value |
| **Sex** |  |  |  |  |  |  |
| *Male* | - | - | - | Ref | Ref |  |
| *Female* | - | - | - | **1.33** | **1.03 – 1.71** | **0.029** |
| **Age** (years) |  |  |  |  |  |  |
| *≥75* | Ref | Ref |  | Ref | Ref |  |
| *65-74* | 1.60 | 0.90 – 2.85 | 0.111 | 1.24 | 0.93 – 1.67 | 0.145 |
| *40-64* | 1.41 | 0.80 – 2.50 | 0.232 | 1.25 | 0.91 – 1.72 | 0.168 |
| *≤39* | 1.52 | 0.82 – 2.83 | 0.184 | **2.37** | **1.50 – 3.73** | **<0.001** |
| **Disease status** |  |  |  |  |  |  |
| *Curable* | Ref | Ref |  | Ref | Ref |  |
| *Incurable* | 1.12 | 0.74 – 1.64 | 0.561 | 0.93 | 0.71 – 1.22 | 0.604 |
| **Education** |  |  |  |  |  |  |
| *Low* | Ref | Ref |  | Ref | Ref |  |
| *Medium* | 1.11 | 0.74 – 1.66 | 0.610 | 1.01 | 0.78 – 1.32 | 0.922 |
| *High* | 1.32 | 0.87 – 1.99 | 0.192 | 0.86 | 0.65 – 1.14 | 0.305 |
| **Receiving treatment** |  |  |  |  |  |  |
| *Yes* | 0.69 | 0.39 – 1.21 | 0.195 | 0.78 | 0.57 – 1.05 | 0.096 |
| **Physical functioning** *(per 10)* | **0.83** | **0.78 – 0.89** | **<0.001** | **0.81** | **0.77 – 0.85** | **<0.001** |
| **Tumour type** |  |  |  |  |  |  |
| *Colorectal* | - | - | - | Ref | Ref |  |
| *Prostate* | - | - | - | 1.27 | 0.85 – 1.88 | 0.245 |
| *Upper GI* | - | - | - | 0.96 | 0.66 – 1.40 | 0.844 |
| *Urology* | - | - | - | 1.15 | 0.75 – 1.76 | 0.519 |
| *Other* | - | - | - | 0.94 | 0.68 – 1.29 | 0.687 |
| **Type of hospital** |  |  |  |  |  |  |
| *General* | Ref | Ref |  | Ref | Ref |  |
| *Teaching* | 1.29 | 0.95 – 1.77 | 0.106 | 0.84 | 0.65 – 1.10 | 0.200 |
| *Academic* | 0.93 | 0.48 – 1.81 | 0.838 | **0.62** | **0.43 – 0.89** | **0.009** |
| **Travel time** (minutes) |  |  |  |  |  |  |
| *<30* | Ref | Ref |  | Ref | Ref |  |
| *30-60* | **2.30** | **1.55 – 3.43** | **<0.001** | **1.77** | **1.35 – 2.32** | **<0.001** |
| *>60* | **5.90** | **1.91 – 18.25** | **0.002** | **2.69** | **1.70 – 4.24** | **<0.001** |
| **Frequency of visits** |  |  |  |  |  |  |
| *Daily or weekly* | Ref | Ref |  | Ref | Ref |  |
| *Every 2-4 weeks* | 1.05 | 0.72 – 1.53 | 0.800 | 0.81 | 0.63 – 1.05 | 0.110 |
| *Every 3 months or less* | 0.64 | 0.34 – 1.18 | 0.153 | 0.87 | 0.59 – 1.28 | 0.476 |
| **Travel mode*** |  |  |  |  |  |  |
| *Independent* | Ref | Ref |  | Ref | Ref |  |
| *Dependent* | 1.21 | 0.88 – 1.67 | 0. 231 | **1.44** | **1.14 – 1.82** | **0.002** |

* Travel mode was defined as independent (on their own: walking, cycling or by car) or dependent (someone else drove them, public transport or taxi services provided by the hospital).

**Appendix 4: Multivariable logistic regression analysis assessing which factors are associated with willingness to travel >30 minutes for oncological treatment in the total population, breast cancer patients only and in all other tumour types except breast cancer.**

|  | **Total population**  **(n=2583)** | | | **Breast cancer**  **(n=925)** | | | **All other tumour types**  **(n=1658)** | | |
| --- | --- | --- | --- | --- | --- | --- | --- | --- | --- |
|  | Odds | 95% CI | p-value | Odds | 95% CI | p-value | Odds | 95% CI | p-value |
| **Sex** |  |  |  |  |  |  |  |  |  |
| *Male* | Ref | Ref |  | - | - |  | Ref | Ref |  |
| *Female* | 1.12 | 0.88 – 1.45 | 0.344 | - | - |  | 1.19 | 0.92 – 1.54 | 0.185 |
| **Age** (years) |  |  |  |  |  |  |  |  |  |
| *≥75* | Ref | Ref |  | Ref | Ref |  | Ref | Ref |  |
| *65-74* | 1.10 | 0.86 – 1.42 | 0.423 | 0.95 | 0.57 – 1.57 | 0.843 | 1.17 | 0.88 – 1.55 | 0.290 |
| *40-64* | 0.95 | 0.73 – 1.22 | 0.677 | 0.74 | 0.46 – 1.19 | 0.216 | 1.08 | 0.80 – 1.47 | 0.606 |
| *≤39* | 0.90 | 0.66 – 1.24 | 0.523 | 0.79 | 0.47 – 1.34 | 0.380 | 0.91 | 0.57 – 1.44 | 0.673 |
| **Disease status** |  |  |  |  |  |  |  |  |  |
| *Curable* | Ref | Ref |  | Ref | Ref |  | Ref | Ref |  |
| *Incurable* | **1.24** | **1.02 – 1.51** | **0.029** | 1.23 | 0.92 – 1.65 | 0.159 | 1.23 | 0.94 – 1.61 | 0.124 |
| **Education** |  |  |  |  |  |  |  |  |  |
| *Low* | Ref | Ref |  | Ref | Ref |  | Ref | Ref |  |
| *Medium* | **1.24** | **1.01 – 1.53** | **0.044** | 1.25 | 0.87 – 1.78 | 0.225 | 1.24 | 0.95 – 1.62 | 0.106 |
| *High* | **1.44** | **1.16 – 1.79** | **0.001** | 1.21 | 0.84 – 1.74 | 0.305 | **1.65** | **1.24 – 2.18** | **0.001** |
| **Tumour type** |  |  |  |  |  |  |  |  |  |
| *Colorectal* | Ref | Ref |  | - | - |  | Ref | Ref |  |
| *Breast* | 0.82 | 0.61 – 1.11 | 0.206 | - | - |  | **-** | **-** |  |
| *Prostate* | 1.32 | 0.92 – 1.89 | 0.127 | **-** | **-** |  | 1.36 | 0.94 – 1.96 | 0.099 |
| *Upper GI* | 1.26 | 0.88 – 1.78 | 0.204 | - | - |  | 1.27 | 0.89 – 1.81 | 0.180 |
| *Urology* | **1.65** | **1.08 – 2.52** | **0.020** | - | - |  | **1.67** | **1.09 – 2.55** | **0.019** |
| *Other* | **1.78** | **1.32 – 2.40** | **<0.001** | **-** | **-** |  | **1.79** | **1.32 – 2.42** | **<0.001** |
| **Physical functioning**  *(per 10)* | **1.13** | **1.08 – 1.17** | **<0.001** | **1.13** | **1.07– 1.20** | **<0.001** | **1.12** | **1.07 – 1.18** | **<0.001** |
| **Travel mode*** |  |  |  |  |  |  |  |  |  |
| *Independent* | Ref | Ref |  | Ref | Ref |  | Ref | Ref |  |
| *Dependent* | 1.13 | 0.95 – 1.36 | 0.171 | 1.29 | 0.97 – 1.71 | 0.078 | 1.02 | 0.81 – 1.29 | 0.834 |

* Travel mode was defined as independent (on their own: walking, cycling or by car) or dependent (someone else drove them, public transport or taxi services provided by the hospital).

**Appendix 5: Multivariable logistic regression analysis assessing which factors are associated with willingness to travel >30 minutes for follow-up in the total population, breast cancer patients only and in all other tumour types except breast cancer.**

|  | **Total population**  **(n=2643)** | | | **Breast cancer**  **(n=927)** | | | **All other tumour types**  **(n=1716)** | | |
| --- | --- | --- | --- | --- | --- | --- | --- | --- | --- |
|  | Odds | Odds | p-value | Odds | 95% CI | p-value | Odds | 95% CI | p-value |
| **Sex** |  |  |  |  |  |  |  |  |  |
| *Male* | Ref | Ref |  | - | - |  | Ref | Ref |  |
| *Female* | 1.24 | 0.97 – 1.58 | 0.092 | - | - |  | **1.35** | **1.05 – 1.74** | **0.021** |
| **Age** (years) |  |  |  |  |  |  |  |  |  |
| *≥75* | Ref | Ref |  | Ref | Ref |  | Ref | Ref |  |
| *65-74* | 1.00 | 0.78 – 1.27 | 0.970 | 1.04 | 0.63 – 1.71 | 0.879 | 0.97 | 0.74 – 1.29 | 0.860 |
| *40-64* | 0.89 | 0.69 – 1.14 | 0.365 | 0.77 | 0.48 – 1.23 | 0.269 | 0.99 | 0.73 – 1.35 | 0.966 |
| *≤39* | 0.92 | 0.67 – 1.27 | 0.612 | 0.86 | 0.51 – 1.45 | 0.582 | 1.00 | 0.62 – 1.62 | 0.986 |
| **Disease status** |  |  |  |  |  |  |  |  |  |
| *Curable* | Ref | Ref |  | Ref | Ref |  | Ref | Ref |  |
| *Incurable* | 1.08 | 0.89 – 1.31 | 0.449 | 1.02 | 0.77 – 1.37 | 0.870 | 1.07 | 0.82 – 1.40 | 0.636 |
| **Education** |  |  |  |  |  |  |  |  |  |
| *Low* | Ref | Ref |  | Ref | Ref |  | Ref | Ref |  |
| *Medium* | **1.39** | **1.13 – 1.71** | **0.002** | 1.22 | 0.86 – 1.74 | 0.266 | **1.53** | **1.18 – 1.99** | **0.001** |
| *High* | **1.75** | **1.41 – 2.18** | **<0.001** | **1.45** | **1.01 – 2.09** | **0.046** | **2.01** | **1.52 – 2.65** | **<0.001** |
| **Tumour type** |  |  |  |  |  |  |  |  |  |
| *Colorectal* | Ref | Ref |  | - | - |  | Ref | Ref |  |
| *Breast* | **0.69** | **0.51 – 0.94** | **0.018** | **-** | **-** |  | **-** | **-** |  |
| *Prostate* | 1.14 | 0.80 – 1.61 | 0.480 | - | - |  | 1.21 | 0.85 – 1.75 | 0.294 |
| *Upper GI* | 1.28 | 0.90 – 1.82 | 0.168 | - | - |  | 1.31 | 0.92 – 1.86 | 0.141 |
| *Urology* | **1.51** | **1.00 – 2.29** | **0.049** | **-** | **-** |  | **1.57** | **1.03 – 2.39** | **0.034** |
| *Other* | **1.64** | **1.22 – 2.21** | **0.001** | **-** | **-** |  | **1.66** | **1.23 – 2.25** | **0.001** |
| **Receiving treatment** |  |  |  |  |  |  |  |  |  |
| *No* | Ref | Ref |  | Ref | Ref |  | Ref | Ref |  |
| *Yes* | 1.02 | 0.79 – 1.32 | 0.874 | 1.16 | 0.70 – 1.92 | 0.559 | 0.96 | 0.71 – 1.30 | 0.794 |
| **Physical functioning**  *(per 10)* | **1.11** | **1.07 – 1.16** | **<0.001** | **1.07** | **1.01 – 114** | **0.046** | **1.15** | **1.10 – 1.20** | **<0.001** |
| **Travel mode*** |  |  |  |  |  |  |  |  |  |
| *Independent* | Ref | Ref |  | Ref | Ref |  | Ref | Ref |  |
| *Dependent* | 1.00 | 0.84 – 1.19 | 0.995 | 1.07 | 0.81 – 1.42 | 0.625 | 0.94 | 0.75 – 1.19 | 0.616 |

* Travel mode was defined as independent (on their own: walking, cycling or by car) or dependent (someone else drove them, public transport or taxi services provided by the hospital).

**Appendix 6: Multivariable logistic regression analysis assessing which factors are associated with willingness to travel >30 minutes for a hospital specialised in their tumour type in the total population, breast cancer patients only and in all other tumour types except breast cancer.**

|  | **Total population**  **(n=2627)** | | | **Breast cancer**  **(n=921)** | | | **All other tumour types**  **(n=1706)** | | |
| --- | --- | --- | --- | --- | --- | --- | --- | --- | --- |
|  | Odds | Odds | Odds | Odds | 95% CI | p-value | Odds | 95% CI | p-value |
| **Sex** |  |  |  |  |  |  |  |  |  |
| *Male* | Ref | Ref |  | - | - |  | Ref | Ref |  |
| *Female* | **1.44** | **1.04 – 2.01** | **0.029** | - | - |  | **1.59** | **1.13 – 2.24** | **0.009** |
| **Age** (years) |  |  |  |  |  |  |  |  |  |
| *≥75* | Ref | Ref |  | Ref | Ref |  | Ref | Ref |  |
| *65-74* | 1.13 | 0.83 – 1.54 | 0.423 | 0.85 | 0.46 – 1.57 | 0.601 | 1.24 | 0.87 – 1.78 | 0.238 |
| *40-64* | 1.13 | 0.82 – 1.56 | 0.463 | 0.74 | 0.41 – 1.33 | 0.310 | 1.46 | 0.97 – 2.19 | 0.071 |
| *≤39* | 0.96 | 0.64 – 1.45 | 0.863 | 0.77 | 0.40 – 1.49 | 0.434 | 0.87 | 0.48 – 1.59 | 0.651 |
| **Disease status** |  |  |  |  |  |  |  |  |  |
| *Curable* | Ref | Ref |  | Ref | Ref |  | Ref | Ref |  |
| *Incurable* | 1.12 | 0.87 – 1.45 | 0.368 | 0.98 | 0.68 – 1.41 | 0.904 | 1.24 | 0.87 – 1.76 | 0.237 |
| **Education** |  |  |  |  |  |  |  |  |  |
| *Low* | Ref | Ref |  | Ref | Ref |  | Ref | Ref |  |
| *Medium* | **1.59** | **1.22 – 2.07** | **0.001** | **1.84** | **1.19 – 2.85** | **0.006** | **1.49** | **1.07 – 2.08** | **0.020** |
| *High* | **1.98** | **1.49 – 2.63** | **<0.001** | **1.75** | **1.12 – 2.73** | **0.012** | **2.29** | **1.57 – 3.35** | **<0.001** |
| **Tumour type** |  |  |  |  |  |  |  |  |  |
| *Colorectal* | Ref | Ref |  | - | - |  | Ref | Ref |  |
| *Breast* | **0.58** | **0.39 – 0.88** | **0.010** | **-** | **-** |  | **-** | **-** |  |
| *Prostate* | 0.96 | 0.60 – 1.52 | 0.859 | - | - |  | 0.98 | 0.61 – 1.57 | 0.921 |
| *Upper GI* | 1.08 | 0.67 – 1.74 | 0.759 | - | - |  | 1.08 | 0.66 – 1.75 | 0.764 |
| *Urology* | 1.27 | 0.72 – 2.23 | 0.415 | **-** | **-** |  | 1.25 | 0.71 – 2.22 | 0.444 |
| *Other* | 1.13 | 0.75 – 1.69 | 0.554 | **-** | **-** |  | 1.14 | 0.76 – 1.72 | 0.521 |
| **Receiving treatment** |  |  |  |  |  |  |  |  |  |
| *No* | Ref | Ref |  | Ref | Ref |  | Ref | Ref |  |
| *Yes* | 0.79 | 0.56 – 1.13 | 0.198 | 1.02 | 0.54 – 1.94 | 0.955 | 0.72 | 0.47 – 1.11 | 0.137 |
| **Physical functioning**  *(per 10)* | **1.14** | **1.09 – 1.19** | **<0.001** | **1.11** | **1.03 – 1.19** | **0.007** | **1.16** | **1.10 – 1.24** | **<0.001** |
| **Travel mode*** |  |  |  |  |  |  |  |  |  |
| *Independent* | Ref | Ref |  | Ref | Ref |  | Ref | Ref |  |
| *Dependent* | 1.09 | 0.87 – 1.38 | 0.454 | 1.35 | 0.95 – 1.93 | 0.097 | 0.90 | 0.66 – 1.23 | 0.514 |

* Travel mode was defined as independent (on their own: walking, cycling or by car) or dependent (someone else drove them, public transport or taxi services provided by the hospital).

**Appendix 7: An English translation of the questionnaire**

**General questions**

|  |  |
| --- | --- |

Date of today: March 2023

1. What is your gender?

❒ Man

❒ Woman

❒ Other

❒ I would rather not say

1. What is your age?

❒ Between 18 and 39 years old

❒ Between 40 and 50 years old

❒ Between 51 and 64 years old

❒ Between 65 and 74 years old

❒ 75 years or older

1. What is your highest education?

*Enter the highest education for which you have a degree*

❒ Lower education

*Example: lower school, LTS, LHNO, domestic school, VMBO, LEAO, ULO, MULO/MAVO and 3 years of HBS*

❒ Medium education

*Example: MBO, MTS, MEAO, HAVO, VWO, HBS, MMS*

❒ Higher education

*Example: HBO and University*

❒ I would rather not say

❒ Other, namely:

1. Are you currently in paid employement?

|  |  |
| --- | --- |

❒ Yes, I work hours per week

❒ Yes, but I am currently on sick leave

❒ No, I am unemployed

❒ Other, namely:

1. What are the first 3 digits of your postcode? (example: 1234 AB = 123)

|  |  |  |
| --- | --- | --- |

**Your illness**

1. Which form of cancer have you (had)?

*Have you (had) multiple cancer types? Then assume the last one.*

❒ Pancreatic cancer

❒ Endometrial cancer

❒ Bladder and other urinary tract cancers

❒ Breast cancer

❒ Colon cancer

❒ Rectal cancer

❒ Ovarian or cervical cancer

❒ Head and/or neck cancer

❒ Skin cancer (no melanoma)

❒ Gastric or oesophageal cancer

❒ Melanoma

❒ Renal cell cancer

❒ Prostate cancer

❒ Sarcoma

❒ Thyroid cancer

❒ Rare form of colon cancer (small intestine, pseudomyxoma peritonei,

Anal cancer)

❒ Other, namely:

1. Are you currently receiving treatment for your cancer?

❒ Yes, treatment with tablets only

❒ Yes, treatment with tablets and medication by infusion or injection

❒ Yes, treatment with medication through infusion or injection only

❒ No, I have yet to start treatment

❒ No, I have never received treatment because it was decided to wait and see how it goes

❒ No, treatment is finished, but I am still getting check-ups in hospital

❒ Other, namely:

1. What is your current disease status?

❒ I (probably) no longer have cancer

❒ I have cancer, but I can get better

❒ I have cancer and cannot get better

❒ My cancer is not going away, but is stable at the moment

❒ I do not know

❒ Other, namely:

1. Are you also under treatment in other hospitals for your cancer, if so how many hospitals?

❒ No, I am only under treatment in this hospital

❒ Yes, in 1 other hospital

❒ Yes, in 2 other hospitals

❒ Yes, in 3 other or more hospitals

**Other diseases and/or conditions**

1. Besides cancer, do you have any other diseases or conditions for which you receive treatment in a hospital?

❒ No, I have no other diseases/conditions 🡪 **continue to question 12 on page 6**

❒ Yes, I have 1 other disease/condition

❒ Yes, I have 2 or more other diseases/conditions

1. Are you receiving treatment at another hospital for these diseases/conditions?

❒ No, I am treated for all my diseases/conditions in this hospital only

❒ Yes, in 1 other hospital

❒ Yes, in 2 other hospitals

❒ Yes, in 3 other or more hospitals

**You choice for this hospital**

1. What were your reasons for choosing this hospital?

*Multiple answers possible (you may tick more than one boxes)*

❒ This hospital is close to home

❒ I already knew this hospital

❒ Through information I found on the internet, this hospital seemed the best for me

❒ My general physician recommended this hospital

❒ Other have recommended this hospital to me

❒ This hospital has a lot of experience in treating my type of cancer

❒ This hospital offered me treatment that I could not get at another hospital

❒ The travel and/or parking costs for this hospital are low

❒ I could get to this hospital quickly / short(er) waiting times

❒ That I can get to hospital by public transport (bus, train, metro, tram)

❒ That I am able to get to hospital with a taxi or a specialised patient transport service

❒ I have no particular reason why I chose this hospital

❒ I do not know / not applicable

❒ Other, namely:

**Travelling to hospital**

1. How often do you have to go to hospital for your cancer? (Think about the past 3 months). *Choose one answer only.*

❒ Almost every day

❒ Every week

❒ Once in 2 weeks

❒ Once in the month

❒ Once in 3 months

❒ Less than once in 3 months

1. Did anyone accompany you to your hospital appointments for your cancer? (Think about the past 3 months) *Multiple answers possible.*

❒ No, I went to hospital alone

❒ Yes, my partner went with me

❒ Yes, my child(ren) went with me

❒ Yes, an acquaintance/ friend(s) went with me

❒ Yes, someone else went with me, namely:

1. How do you usually travel to hospital for your cancer appointments? (Think about the past 3 months). *Choose one answer only.*

❒ I walk to hospital

❒ I cycle to hospital

❒ I drive to hospital by car

❒ Someone else takes me to hospital by car

❒ I take public transport to hospital (bus, train, tram, metro)

❒ I take a taxi or specialised patient transport service to the hospital

❒ Other, namely:

1. On average, how long does it take you to travel to this hospital (one-way)? Think about the past 3 months).

|  |  |
| --- | --- |

|  |  |
| --- | --- |

hours and minutes

1. How do you feel about travelling to the hospital for your cancer treatment or check-up? (Think about the past 3 months). *Choose one answer only.*

❒ I do not find travelling a problem **--> continue to question 19 on page 8**

❒ I sometimes find travelling a problem

❒ I often find travelling problem

❒ I always find travelling a problem

1. Why do you find travelling to the hospital (sometimes) a problem?

*Multiple answers possible (you can tick multiple boxes)*

❒ I do not have my own transport

❒ I find it a burden for people travelling with me

❒ I am (sometimes) too sick or in too much pain to travel

❒ I have to go to hospital very often

❒ I find the travel distance or travel time too long

❒ I find the travel and/or parking costs too high

❒ I cannot go alone and (sometimes) struggle with finding someone to travel with me

❒ I (sometimes) have trouble with the taxi or specialised patients transport service (e.g. taxi arrives late or has to pick up other people)

❒ I need to take time off work

❒ Other namely:

**Willingness to travel longer if necessary**

1. How long at most are you willing to travel for the treatment you are currently receiving (infusion/injections/tablets) (one-way)?

❒ Less than half an hour

❒ Between half an hour and 1 hour

❒ Between 1 hour and 1.5 hours

❒ Between 1.5 and 2 hours

❒ Between 2 and 3 hours

❒ More than 3 hours

❒ I am not receiving treatment now

1. What is the maximum time you are willing to travel for a check-up (no treatment) (one-way)?

❒ Less than half an hour

❒ Between half an hour and 1 hour

❒ Between 1 hour and 1.5 hours

❒ Between 1.5 and 2 hours

❒ Between 2 and 3 hours

❒ More than 3 hours

1. If your treatment and check-ups are at a hospital that is more experienced in your type of cancer, how long are you willing to travel then? (one-way)

❒ Less than half an hour

❒ Between half an hour and 1 hour

❒ Between 1 hour and 1.5 hours

❒ Between 1.5 and 2 hours

❒ Between 2 and 3 hours

❒ More than 3 hours

1. I find the following thing important to take into consideration when deciding upon a maximum travel time for my cancer treatment:

|  |  | Not at all | A little | Quite a bit | Very much |
| --- | --- | --- | --- | --- | --- |
| 1. | That the hospital is close to my home | ❒ | ❒ | ❒ | ❒ |
| 2. | How often I have to visit the hospital | ❒ | ❒ | ❒ | ❒ |
| 3. | That the hospital offers me a treatment that I cannot get at another hospital | ❒ | ❒ | ❒ | ❒ |
| 4. | That the hospital is more experienced in my type of cancer | ❒ | ❒ | ❒ | ❒ |
| 5. | That I can get to the hospital by public transport (bus, train, metro etc.) | ❒ | ❒ | ❒ | ❒ |
| 6. | The travel costs | ❒ | ❒ | ❒ | ❒ |
| 7. | That someone can accompany me to the hospital | ❒ | ❒ | ❒ | ❒ |
| 8. | How I feel (on that day). (e.g. tired or ill) | ❒ | ❒ | ❒ | ❒ |
| 9. | That it fits into my work schedule | ❒ | ❒ | ❒ | ❒ |
| 10. | That I can take part in a study involving a new treatment | ❒ | ❒ | ❒ | ❒ |
| 11. | Other, namely: | ❒ | ❒ | ❒ | ❒ |

We are interested in some things about your health. Please answer all the questions yourself by choosing the answer that best applies to you. There are no "right" or "wrong" answers.

**Your health**

1. During the past week:

|  |  | Not at all | A little | Quite a bit | Very much |
| --- | --- | --- | --- | --- | --- |
| 1. | Did you have trouble doing strenuous activities, like carrying a heavy shopping bag or suitcase? | ❒ | ❒ | ❒ | ❒ |
| 2. | Did you have trouble taking a long walk? | ❒ | ❒ | ❒ | ❒ |
| 3. | Did you have trouble taking a short walk outside the house? | ❒ | ❒ | ❒ | ❒ |
| 4. | Did you need to stay in bed or a chair during the day? | ❒ | ❒ | ❒ | ❒ |
| 5. | Did you need help with eating, dressing, washing yourself or using the toilet? | ❒ | ❒ | ❒ | ❒ |

1. Do you have any other comments or remarks following this questionnaire?
